# Prioritizing Nutrition in Medical Education—the Time Has Come

**DOI:** 10.1016/j.advnut.2024.100231

**Published:** 2024-05-07

**Authors:** Andrew A Bremer

**Affiliations:** Office of Nutrition Research, National Institutes of Health, Bethesda, MD, United States

The importance of nutrition in optimizing health has been recognized for millennia, as has the concept of “food is medicine.” In fact, Hippocrates may be credited for starting the “food is medicine” movement around 400 BC with his attributed quote, “Let food be thy medicine, and medicine be thy food.” However, despite advances in myriad areas of medicine and public health, significant gaps remain in our understanding of why and how food and nutrition affect health [1]. Unfortunately, significant gaps also remain in medical education with respect to nutrition education and our understanding of the importance of nutrition on health.

As highlighted in their article, “There and Back Again: A 40-Year Perspective on Physician Nutrition Education,” Albin et al. [2] stress the urgent need for improved evidence-based physician nutrition education, especially given that the United States and indeed the whole world are currently facing an urgent nutrition-related health crisis. The authors note how little has changed with respect to incorporating nutrition education into medical school curricula since the publication of the 1985 National Academies report “Nutrition Education in U.S. Medical Schools” and how most medical schools still fail to include the recommended minimum of 25 hours of nutrition training in their undergraduate medical education (UME). Without foundational concepts of nutrition in UME, Albin et al. also note how graduate medical education (GME) unsurprisingly falls short of meeting patient needs for nutritional guidance in clinical practice. Furthermore, the authors highlight the May 2022 bipartisan resolution in the United States House of Representatives [introduced by Representatives James McGovern (D-MA) and Michael Burgess (R-TX)] calling for meaningful nutrition education in medical schools, residency and fellowship programs, and other health professional training programs [3], as well as the subsequent Summit on Medical Education in Nutrition hosted by the Accreditation Council for GME, the Association of American Medical Colleges, and the American Association of Colleges of Osteopathic Medicine which was held to focus on strategies to advance nutrition education across the medical education continuum [4].

Albin et al. [2] further outline numerous strategies to integrate nutrition into medical education pathways—including the emerging field of culinary medicine, an evidence-based field combining the culinary arts with nutritional science and medicine—as well as the recent recognition and adoption of Food is Medicine (FIM) programs across the country. The authors then conclude that advancing the critical need for widespread adoption of nutrition education for physicians will require support and engagement across societal stakeholders, including co-leadership from registered dietitian nutritionists (RDNs), health system and payor reform, and opportunities for clinical innovation. The Office of Nutrition Research (ONR) at the NIH thank the authors for their thorough manuscript and is supportive of efforts to strengthen evidence-based approaches in this space.

The ONR at NIH is very engaged in these issues, especially because: *(i)* suboptimal diets are responsible for more deaths than any other risk factor globally (including tobacco smoking, air pollution, and high blood pressure) [5]; *(ii)* malnutrition—in all of its forms—is the leading cause of morbidity and mortality in the world [6]; and *(iii)* 7 of the 10 leading causes of death in the United States are directly affected by diet [7]. Moreover, because diet directly plays a key role in the pathogenesis of many chronic diseases (including but not limited to obesity, cardiovascular disease, hypertension, stroke, type 2 diabetes, some cancers, and perhaps some neurological diseases), ONR recognizes that dietary interventions, in conjunction with RDN involvement and social support, have the ability to vastly improve population health and reduce health care costs [8]. Furthermore, ONR is committed to advancing research in the field of FIM [9] and has been actively engaged in the United States Department of Health and Human Services FIM activities [10] as well as promoting FIM research among the 27 Institutes and Centers at the NIH. Moreover, one of the panels at the 2022 White House Conference on Hunger, Nutrition, and Health was “Food is Medicine: Bringing nutrition out of the health care shadows” [11], and the FIM paradigm is a key element within the National Strategy on Hunger, Nutrition, and Health [12].

As stated previously by my colleague, “imagine a world where greater attention is paid to the power of nutrition in healthcare” [9]. Wouldn’t that be wonderful? And that starts with education—both of medical trainees and practicing physicians. To close, Albin et al. [2] present an opportunity for thoughtful innovation and change, emphasizing key curricular areas in medical education including: *(i)* a multi-modal, longitudinal, interprofessional approach to medical nutrition education; *(ii)* core collaborations with RDNs, speech pathologists, physical and occupational therapists, social workers, pharmacists, behavior change experts, and other colleagues; *(iii)* nutrition education including more than basic biochemistry but rather also addressing real life scenarios; *(iv)* the relevance of food policies in determining equitable access to healthy food; and *(v)* the importance of primary care payment reforms and value alignment, including physician compensation for delivering health education, to holistic patient care.

The time to prioritize nutrition in medical education is now. ONR is supportive in these efforts and will continue its mission to advance nutrition science research to promote health across the lifespan and to support the development of evidence-based, equitable, context-specific, culturally appropriate, resilient, and sustainable solutions to reduce the burdens of diet-related diseases and health disparities.

## Author contributions

The sole author was responsible for all aspects of this manuscript.

## Conflict of interest

The contents of this article represent the author’s views and do not constitute an official position of the National Institutes of Health or the United States Government. The author is an Editor of *Advances in Nutrition* and played no role in the Journal’s evaluation of the manuscript.
